# The Prevalence of Online Health Information Seeking Among Patients in Scotland: A Cross-Sectional Exploratory Study

**DOI:** 10.2196/resprot.4010

**Published:** 2015-07-15

**Authors:** Julia Moreland, Tara L French, Grant P Cumming

**Affiliations:** ^1^ University of the Highlands and Islands Moray College Elgin United Kingdom; ^2^ University of Aberdeen Aberdeen United Kingdom; ^3^ National Health Service (NHS) Grampian Elgin United Kingdom

**Keywords:** online health information seeking, health care-seeking behavior, health information seeking, health seeking, digital divide

## Abstract

**Background:**

Online health information seeking is an activity that needs to be explored in Scotland. While there are a growing number of studies that adopt a qualitative approach to this issue and attempt to understand the behaviors associated with online health information seeking, previous studies focusing on quantifying the prevalence and pattern of online health seeking in the United Kingdom have been based on Internet users in general.

**Objective:**

This exploratory study sought to describe the prevalence of online health information seeking in a rural area of Scotland based on primary data from a patient population.

**Methods:**

A survey design was employed utilizing self-completed questionnaires, based on the Pew Internet and American Life Project; questionnaires were distributed among adult patients in 10 primary care centers in a rural community in Scotland.

**Results:**

A convenience sample of 571 (0.10% of the total population in Grampian, N=581,198) patients completed the questionnaire. A total of 68.4% (379/554) of patients had previously used the Internet to acquire health information. A total of 25.4% (136/536) of patients consulted the Internet for health information regarding their current appointment on the day surveyed; 34.6% (47/136) of these patients were influenced to attend their appointment as a result of that online health information. A total of 43.2% (207/479) of patients stated the health information helped improve their health and 67.1% (290/432) indicated that they had learned something new. A total of 34.0% (146/430) of patients talked to a health professional about the information they had found and 90.0% (376/418) reported that the information was useful. In total, 70.4% (145/206) of patients were concerned about obtaining health information online from reliable sources. A total of 67.1% (139/207) of patients were concerned that a health site may sell their personal information, yet only 6.7% (36/535) checked the privacy policy of the site visited. However, 27.9% (55/197) of patients were not concerned about their employer finding out what health sites they visited, whereas 37.5% (78/208) were concerned that others would find out.

**Conclusions:**

The results suggest that online health information-seeking behavior influences offline health-related behavior among the population surveyed. Patient attitudes to online health information seeking were focused on issues relating to trust, reliability, privacy, and confidentiality. This study provides support for the growing phenomenon of an empowered, computer-literate, health information consumer, and the impact of this phenomenon must be considered in the context of the patient-health professional dynamic. The unpredictable nature of human thought and action in relation to this field of study requires an ongoing program of ethnographic research, both physical and virtual, within a Health Web Science framework. This study has provided a baseline of the prevalence of online health information seeking in the Grampian region of Scotland.

## Introduction

### Overview

The Scottish Government recently published a National Framework [1] to encourage digital participation at a local level in the hope that the Scottish people would have the opportunity to benefit from the wide range of information, goods, and services to which the Internet provides access. The Scottish Government are particularly focused on improving digital participation among those groups who historically have been less likely to access the Internet, such as the elderly and low-income households [2]. Paradoxically, these groups stand to benefit most from reduced-price goods and other lifestyle benefits, which the Internet can provide. Internet use at home has been steadily increasing in Scotland. From 2007 to 2013 the percentage of adults accessing the Internet for personal use has risen 17.1 points (62.7% in 2007 to 79.8% in 2013) [2]. This compares with a 15-point increase of Internet use among adults in the United States for the same period (71% in 2007 to 86% in 2013) [3]. Internet use in the home is increasing and, therefore, a range of online activities are potentially impacting people’s lives. Information relating to health and well-being is one area in which the Internet is becoming increasingly important.

Scotland has a health service that is free at the point of need. As the population increases, the health of the nation continues to be an area of concern for the Scottish Government and the National Health Service (NHS) in Scotland. Internet use has enabled patients to access search engines, online symptom checkers, and health information sites to contribute to positive health outcomes for themselves or a loved one. This digitally literate population are described as "health seekers" [4]. Humans have information needs, which lead to certain behaviors in order to meet these needs [5]. Information seeking is both a conscious and unconscious process, which encompasses how information is sought, found, used, and also avoided [5]. Seeking information can fill gaps in knowledge and, therefore, decreases anxiety, which in itself can influence and improve health outcomes [6].

Online health seekers differ from offline health seekers by age, income, and education [7]. Those accessing health information online are affluent, well-educated adults [8]. These differences contribute to what is known as the digital divide [9-11]. In 2011, the US government launched a 38 million dollar scheme aimed at narrowing the digital divide, recognizing that health information online is contributing positively to health outcomes and that those who are medically disadvantaged need support in accessing the Internet [12].

Health information sought online by patients is not intended to replace physician care, but rather, to support it [13]. Health professionals also seek information online to help with a diagnosis and provide reassurance [13]. Health seekers are often looking for information about a loved one's care, which suggests a caregiver role [13]. Reasons for health seeking include the following: doing what is prescribed (professional logic), using personal judgement to inform decisions (consumer logic), and gathering information and experience from others (community logic) [13]. Through analysis of the patterns of online health seekers and these forms of logic there is a balance of power to be negotiated between patient and health care professional. Consumer and community logic can often draw credibility from health forums, which are largely user generated. This may create tension in the dynamic between patient and health professional which requires both parties to renegotiate traditional roles, for instance, health professionals are not the only source of health information for patients. Furthermore, online information and social support is encouraging patients not to adhere to physician advice [14], to which health care professionals must adapt [15].

The online health seeker expects convenience, to be a partner in decision making, and almost instant service in all aspects of their health care [16]. However, the health seeker must pass through a series of complex processes in order to access and utilize health information [6]. Barriers in language, information and communication technologies (ICTs) knowledge, or the ability to weigh up sources and formulate a reasoned perspective can all limit the positive outcomes of health seeking online. Trust has also been identified as a key barrier to improving the online health information-seeking experience [17].

Making a primary care appointment can be highly bureaucratized, often with considerable waiting times. Access to primary care for patients within the United Kingdom is guaranteed within 48 hours of contacting their general practitioner (GP) [18]. In Scotland, 29% of adult patients reported that they can wait up to a week for an appointment [19]. However, time constraints, busy lives, and anxiety about symptoms mean that when it comes to medical advice, people want it here and they want it now. Not only do they want instantly available advice, 75% of consumers want access to monitoring devices and online tools [20], thereby allowing visits to the doctor, or other health professional, to be reduced.

The point of origin for this initial exploratory study is concerned with the effect of online health information on primary care services in Scotland and the potential impact this has on the doctor-patient relationship. As stated previously, health seekers often want to be partners in their health care; as Ball and Lillis [16] stated:

The empowered, computer-literate public is exerting tremendous influence on healthcare delivery. Consumer interest in and demand for online administrative processes, information-rich Internet health portals, and access to physician web pages and e-mail has introduced a new dimension to maintaining wellness and treating disease.

### Objective

There is a growing body of literature that is concerned with the prevalence and patterns of online health information seeking. This study hopes to contribute to that body of knowledge by providing an overview of online health information seeking among a patient population in Scotland. It is hoped that this information will create a baseline indicating what is happening in relation to online health information seeking, rather than an explanation of patient motivation to do so. By simply discovering what is happening, for instance, how many and who, the authors hope to provide a starting point for future research focused on finding out what motivates online health seekers and gain a deeper understanding of the behavior involved. The findings of this study will also be useful to health policy makers and health website content regulators.

## Methods

### Overview

This study involved a cross-sectional survey and data were gathered using a self-completed questionnaire. The questionnaire was adapted from the Pew Internet and American Life Project [4] (see Multimedia Appendix 1 for the full text of the questionnaire). Questions were closed response and patients were asked to select one response from a range of categorical options. Additional questions were inserted in order to gather data on whether online health information was sought prior to the patient’s current appointment and the importance of privacy, convenience, and confidentiality to the patient. The questionnaire was pilot-tested on a sample of students attending the University of the Highlands and Islands (UHI).

The timetable for this cross-sectional study was at the health care center manager's discretion, but no more than one calendar month as per ethical approval. A total of 800 questionnaires were distributed to 10 medical centers in Moray, Scotland, and yielded a response rate of 71.4% (571/800). This sample of 571 patients represented 0.10% of the total population in Grampian (N=581,198 [21]).

### Respondents

All adult patients (aged 18 years and over) attending the medical center were invited to participate. The definition of a patient within this study is a person who is attending a medical center on the day surveyed. Table 1 indicates the demographic data from patients who completed the questionnaire.

### Procedure

Questionnaires were placed in health care centers with the permission and ethical approval of NHS Scotland, the National Research Ethics Service (NRES), and the University of the Highlands and Islands. Health care center personnel handed the questionnaires out to patients as they waited to see a health professional. A study brief and consent form were read and completed prior to the patient completing the questionnaire to ensure that only adult patients responded.

Anonymized self-completed questionnaires were deposited in a post box in the medical center and collected by a researcher. The software program Statistical Package for the Social Sciences (SPSS) version 22 was used to perform statistical analysis. As the data were nominal, nonparametric tests were employed. Chi-square tests with 95% confidence levels were used to identify differences among the patient demographics. Cramer’s V and phi were calculated to indicate the presence and strength of any relationship between variables, as even though the confidence interval is high, the strength of the effect is only indicated by the appropriate coefficient (phi for 2x2 tables or Cramer’s V for larger tables).

**Table 1:** Demographic data of patients surveyed in Moray, Scotland (n=571).

Characteristics		Frequency, n (%)
**Age groups (in years)**		
	18-25	82 (14.4)
	26-35	89 (15.6)
	36-45	107 (18.7)
	46-55	94 (16.5)
	56-65	88 (15.4)
	66-75	51 (8.9)
	76-85	15 (2.6)
	86+	2 (0.4)
	Not completed	43 (7.5)
**Gender**		
	Male	186 (32.6)
	Female	325 (56.9)
	Not completed	60 (10.5)
**Employment status**		
	Full-time paid employment	246 (43.1)
	Part-time paid employment	71 (12.4)
	Full-time student	24 (4.2)
	Part-time student	3 (0.5)
	Home duties	26 (4.6)
	Retired	122 (21.4)
	Unemployed	19 (3.3)
	Caregiver	2 (0.4)
	Ill	1 (0.2)
	Not completed	36 (6.3)
**Educational attainment**		
	No formal qualification	52 (9.1)
	Standard Grade, "O" grade	102 (17.9)
	Highers	75 (13.1)
	Vocational qualification	73 (12.8)
	Higher National Diploma	2 (0.4)
	Undergraduate degree	63 (11.0)
	Masters	18 (3.2)
	Doctorate	1 (0.2)
	Professional qualification	98 (17.2)
	Not completed	70 (12.3)
**Location**		
	Rural	90 (15.8)
	Village	172 (30.1)
	Town	200 (35)
	City	69 (12.1)

## Results

### Demographics and Prevalence of Online Health Information Seeking

Table 1 presents the demographic data gathered on the patients who responded to the survey and Table 2 contains the patient responses to the survey. A total of 554 patients responded to a question asking if they had previously searched for health information on the Internet. Of these responses, 379 (68.4%) had previously searched for health information online, with 63.7% (353/554) of patients doing this by themselves and 4.7% (26/554) doing so on behalf of someone else.

Furthermore, 25.4% (136/536) of patients had consulted the Internet for health information in relation to their appointment on the day surveyed. This was either by themselves—21.5% (115/536)—or someone had done so on their behalf—3.9% (21/536). Of the patients who consulted the Internet for health information prior to their current appointment, 34.6% (47/136) stated that the information they had found online had influenced them to attend their current appointment and 15.4% (53/344) also indicated that they would not have otherwise attended the current appointment.

A large proportion of patients (211/483, 43.7%) stated that they had found information online which had helped them to improve their health, and 90.0% (376/418) believed the health information that they found online was useful. Tables 3 and 4 present the channels and specific sites used. There was a very low response rate (16/571, 2.8%) to specific sites used by the patient sample; this may have been due to issues with memory recall. Table 3 shows that almost half of the patients (53/108, 49.1%) used a search engine, 33.3% (36/108) used NHS sites, and 17.6% (19/108) used a health forum.

The age range dispersion between 18 to 65 years was relatively equal given that opportunity sampling was employed. Patients who took part in the survey were predominantly female (325/571, 56.9%), in full-time employment (246/571, 43.1%) or retired (122/571, 21.4%), and educated to Standard Grade or Highers (177/571, 31.0%)—equivalent to Advanced Subsidiary (AS) level in England and Northern Ireland—which is a reflection of general survey response bias [22]. The classification of patient home location was a relatively even divide between rural and urban. However, it should be noted that the geographical location of this study is generally classified as being a rural area.

**Table 2:** Patient responses to the survey.

Questions and responses		Frequency, n (%)
**Q1. Have you or someone acting on your behalf previously used the Internet to look up health information? (n=554)**		
	Yes, myself	353 (63.7)
	Yes, someone on my behalf	26 (4.7)
	No	175 (31.6)
	Total	554 (100)
**Q4. Did you or someone acting on your behalf search for health information recently with regard to your current appointment? (n=536)**		
	Yes, myself	115 (21.5)
	Yes, someone on my behalf	21 (3.9)
	No	400 (74.6)
	Total	536 (100)
**Q6. Did the health information influence your decision to attend your appointment today? (n=398)**		
	Yes	47 (11.8)
	No	351 (88.2)
	Total	398 (100)
**Q7. Would you have attended this medical center today if you had not found this information? (n=345)**		
	Yes	292 (84.6)
	No	53 (15.4)
	Total	345 (100)
**Q8. Have you previously found information on the Internet which has helped you improve your health? (n=483)**		
	Yes	211 (43.7)
	No	272 (56.3)
	Total	483 (100)
**Q9. Overall, how useful was the health information you got online? (n=418)**		
	Useful	376 (90.0)
	Not useful	42 (10.0)
	Total	418 (100)
**Q10. Did you talk to a health professional about the information you got online? (n=430)**		
	Yes	146 (34.0)
	No	284 (66.0)
	Total	430 (100)
**Q11. Did you learn anything new from the information you got online? (n=432)**		
	Yes	290 (67.1)
	No	142 (32.9)
	Total	432 (100)

**Table 3:** Channels used by patients to search for health information online (n=108).

Channels	Frequency, n (%)
Health forum	19 (17.6)
Search engine	53 (49.1)
National Health Service website	36 (33.3)
Total	108 (100)

**Table 4:** Specific sites used by patients to search for health information online (n=16).

Specific sites used	Frequency, n (%)
BBC.co.uk [23]	1 (6)
Menopausematters.co.uk [24]	1 (6)
Boots.com [25]	1 (6)
CDC.gov [26]	1 (6)
Public Health England (GOV.UK) [27]	1 (6)
Fibromyalgia.co.uk [28]	1 (6)
Google [29]	5 (31)
JustAnswer.co.uk [30]	1 (6)
NHS24.com/SelfHelpGuide [31]	1 (6)
Patient.co.uk [32]	3 (19)
Total	16 (100)

### Characteristics That Influence Online Health Information Seeking

As shown in Table 5, a weak association was found between employment status and those who previously searched for health information online. Results also revealed a weak association between employment status and those who were influenced to attend the current appointment as a result of online health information. There was no association between employment status and online health information seeking prior to the patient's appointment on the day surveyed.

A weak association is evident between educational attainment and those who previously searched for health information online. A weak association was also found between educational attainment and those who were influenced to attend the current appointment as a result of online health information. There was no association between educational attainment and online health information seeking prior to the patient's appointment on the day surveyed.

There was a weak association between location and those who previously searched for health information online. A weak association was also found between location and those who were influenced to attend the current appointment as a result of online health information. There was no association between location and online health information seeking prior to the patient's appointment on the day surveyed.

There was a weak association between age and those who had previously sought health information online. There was also a weak association between age and those patients who were influenced to attend the appointment as a result of online health information. There was no association between online health information seeking prior to the appointment on the day surveyed and age.

A weak association was found between gender and online health seeking prior to the patient's appointment on the day surveyed. There was a weak association between gender and those patients who were influenced to attend the appointment as a result of online health information.

As shown in Table 6, a large proportion of patients (357/486, 73.5%) indicated that getting information online, as opposed to other sources, was important. Patients were also concerned about their employer finding out which health sites they visited (55/197, 27.9%), reliability of sources (145/206, 70.4%), and the security of information of their online searches (139/207, 67.1%). However, in relation to concern for security, only a small proportion of patients (36/535, 6.7%) checked the website privacy policy in relation to how their data may be used.

**Table 5:** Effect of socioeconomic characteristics on health-seeking behavior.

Socioeconomic characteristic and health-seeking behavior effect		χ^2^(df)	*P*	Cramer's V or phi
**Does employment status have an effect on online health-seeking behavior?**				
	Patients who previously searched for online health information (n=554)	23.2 (9)	<.001	.21 (V)
	Patients influenced to attend current appointment (n=571)	89.9 (27)	<.001	.23^a^(V)
	Patients who searched prior to current appointment (n=536)	10.3 (9)	.33	N/A^b,c^
**Does educational attainment have an effect on online health-seeking behavior?**				
	Patients who previously searched for online health information (n=554)	21.0 (9)	<.001	.36 (V)
	Patients influenced to attend current appointment (n=571)	76.0 (12)	<.001	.21 (V)
	Patients who searched prior to current appointment (n=536)	4.9 (4)	.29	N/A^c^
**Does location have an effect on online health-seeking behavior?**				
	Patients who previously searched for online health information (n=554)	21.0 (4)	<.001	.20 (V)
	Patients influenced to attend current appointment (n=771)	76.0 (12)	<.001	.21 (V)
	Patients who searched prior to current appointment (n=536)	4.9 (4)	.29	N/A^c^
**Does age have an effect on online health-seeking behavior?**				
	Patients who previously searched for online health information (n=554)	52.3 (8)	<.001	.31 (V)
	Patients influenced to attend current appointment (n=571)	94.0 (24)	<.001	.23 (V)
	Patients who searched prior to current appointment (n=536)	7.7 (8)	.47	N/A^c^
**Does gender have an effect on online health-seeking behavior?**				
	Patients who previously searched for online health information	N/A	N/A	N/A
	Patients influenced to attend current appointment (n=571)	46.5 (6)	<.001	.29 (phi)
	Patients who searched prior to current appointment (n=554)	35.1 (2)	<.001	.25 (phi)

^a^A total of 20 cells were expected to have a count less than 5.

^b^Not applicable (N/A).

^c^Too many cells violated the expected count.

**Table 6:** Attitudes evident among patients in relation to online health information seeking.

Attitudes	n (%)
Concerned that their employer might find out what health sites they visited	55/197 (27.9)
Concerned about getting health information from an unreliable source	145/206 (70.4)
Felt it was important that they could get health information online rather than from other sources	357/486 (73.5)
It is important to be able to get health information online anonymously without having to talk to anyone	357/505 (70.7)
It is important that they can get health information online at any time	433/509 (85.1)
Not concerned about other people finding out what health sites they have visited (1 out of 10 patients were very concerned about this)	130/208 (62.5)
Concerned that a website might sell or give away information about what they did online	139/207 (67.1)
Checked the health or medical website's privacy policy to read about how the site uses personal information	36/535 (6.7)

## Discussion

### Principal Findings

Patients are searching for health information online and this information influences a small proportion to attend medical centers. Attitudes to online health information seeking suggest a concern for reliability, convenience, privacy, and a preference for online health information above other sources. These results also indicate that location, age, and gender do have an effect on the prevalence of online health information-seeking behavior and the resulting offline behavior.

The Pew Project [7] suggests a slightly higher proportion of Americans are using the Internet to search for health information (80% in 2011). The Oxford Internet Survey (OxIS) [9] reported this figure as 71% in 2011 and falling to 69% in 2013 [10], which is closer to the findings of this study at 68.4%. Cultural differences and a health care service that is not free at the point of need in the United States may explain the difference between the United Kingdom's and the United States' levels of health information seeking.

This study suggests that online health information seeking influences the offline behavior of this patient sample through patients consulting the Internet for health information either by themselves or on behalf of someone else. Some patients have been influenced to attend a medical center as a direct result of information they found online, with a small proportion of patients reporting that they would not have attended the appointment without this information.

Almost all of the patients in this sample population stated that they found online health information useful. Two-thirds of patients claimed they had not discussed this information with a health professional and the same proportion of patients indicated that they had learned something new from the online health information. It could be suggested that patients who consult the Internet for health information and are satisfied may not feel the need to then consult a health professional. However, further research is needed to explore this finding and investigate whether health professionals are being bypassed by patient online health information seeking. As the Web evolves and attitudes to the Web change, this research needs to be ongoing.

The results from this primary data suggest that location, age, and gender have an effect on health-seeking behaviors and the resulting actions, but the association is weak. In line with the Pew Project [7] and OxIS [9,10], large proportions of those surveyed are searching for health information online. However, it must be highlighted that the findings of the Pew Project and OxIS were both taken from secondary sources of data concerned with overall behavior of Internet users. Health information is influencing patients in Moray to attend medical centers when they would not have done so without the information. Therefore, this study sets a precedent for establishing baseline data for online health-seeking activities among patient populations.

The findings provide support that the digital divide has an impact on health information seeking [11]. In this study, a weak association was found between location and health information seeking. However, the geographical area in this study is considered as a predominantly rural area and, therefore, the self-reported location of patients cannot be truly classified as being within a true rural/urban setting. A national-level study is needed to provide primary data, which would investigate the occurrence of online health information seeking among patients across Scotland focusing on the rural/urban dichotomy.

Confidentiality and privacy is important to patients when they search for health information online, especially in relation to how their personal information may be used and the privacy of their search content. In this instance, "others" are a concern when it comes to people finding out about the content of searches and employers are not a concern. This may suggest that online health information seeking is not taking place at work, however, this would require further research.

The implications of online health information-seeking behavior on the power dynamic of the traditional health professional and patient relationship should also be the subject of future research as a result of the public availability via the Internet of previously exclusive information (ie, medical information for professionals only). For example, change in the power dynamic because knowledge of the health professional is becoming democratized may cause issues around treatment adherence based on trust and the value that patients place on the knowledge of health professionals.

### Conclusions

The findings of this survey provide an indication of how patients' offline behavior is influenced by health information they find online. This study has provided support for the findings from secondary data of previous research which showed that a large number of people are accessing the Internet for health information. This study’s unique contribution lies in its presentation of evidence based on primary data, which quantifies patients who are influenced by online health information to interact with health care professionals by attending medical centers. This phenomenon needs to be considered in the context of individual countries and specific populations in order to be useful to policy makers.

Further research is needed to evaluate the impact that the democratization of medical information through online health information seeking among patients has on health care professionals and organizations, including how to access those who sought health information online and did not attend a medical center as a result. Patients want access to health information online at any time, in preference to other sources, and this may be related to increased anonymity and privacy.

The numbers of online health information-seeking patients are increasing; health care professionals and their supporting organizations need to consider how to respond to this. With the increasing amount of user-contributed health information, consideration must be given as to the provision of online health information for digital natives versus digital immigrants, for instance, those who have been socialized in a culture in which digital technologies are part of everyday life compared to those who have had to develop an understanding of digital technologies as adults.

This study provides support for the growing phenomenon of an empowered, computer-literate, health information consumer and the impact of this phenomenon must be considered in the context of the patient-health professional dynamic. The unpredictable nature of human thought and action in relation to this field of study requires a program of ethnographic research, both physical and virtual, to describe how people use the Web for health.

## Abbreviations

AS: Advanced Subsidiary
ICT: information and communication technology
GP: general practitioner
N/A: not applicable
NHS: National Health Service
NRES: National Research Ethics Service
OxIS: Oxford Internet Survey
SPSS: Statistical Package for the Social Sciences
UHI: University of the Highlands and Islands

## Appendix

Multimedia Appendix 1 Full text of the self-completed questionnaire.

## References

[1] (2014). Digital Participation: A National Framework for Local Action.

[2] (2014). Scotland's People Annual Report: Results from 2013 Scottish Household Survey.

[3] (2014). Pew Research Center.

[4] Rainie L, Fox S (2000). Pew Research Center.

[5] Case DO (2012). Looking for Information: A Survey of Research on Information Seeking. 3rd edition.

[6] Johnson JD, Case DO (2012). Health Information Seeking.

[7] Fox S (2011). Pew Research Center.

[8] Hargittai E (2004). Internet access and use in context. New Media and Society.

[9] Dutton WH, Blank G (2011). Next Generation Users: The Internet in Britain.

[10] Dutton WH, Blank G, Groselj D (2013). Cultures of the Internet: The Internet in Britain.

[11] Cotten SR, Gupta SS (2004). Characteristics of online and offline health information seekers and factors that discriminate between them. Soc Sci Med.

[12] Lustria ML, Smith SA, Hinnant CC (2011). Exploring digital divides: an examination of eHealth technology use in health information seeking, communication and personal health information management in the USA. Health Informatics J.

[13] Ybarra ML, Suman M (2006). Help seeking behavior and the Internet: a national survey. Int J Med Inform.

[14] Weaver JB 3rd, Thompson NJ, Sargent Weaver S, Hopkins GL (2009). Healthcare non-adherence decisions and Internet health information. Comput Human Behav.

[15] Lober WB, Flowers JL (2011). Consumer empowerment in health care amid the Internet and social media. Semin Oncol Nurs.

[16] Ball MJ, Lillis J (2001). E-health: transforming the physician/patient relationship. Int J Med Inform.

[17] Sillence E, Briggs P (2007). Please advise: using the Internet for health and financial advice. Comput Human Behav.

[18] Godden S, Pollock AM (2009). Waiting list and waiting time statistics in Britain: a critical review. Public Health.

[19] (2013). PHA Media Results.

[20] (2008). 2008 Survey of Health Care Consumers: Executive Summary.

[21] (2014). ISD Scotland.

[22] Curtin R, Presser S, Singer E (2005). Changes in telephone survey nonresponse over the past quarter century. Public Opin Q.

[23] BBC.

[24] Menopause Matters.

[25] Boots.

[26] Centers for Disease Control and Prevention (CDC).

[27] GOV.UK.

[28] Fibromyalgia.co.uk.

[29] Google.

[30] JustAnswer.

[31] NHS 24.

[32] Patient.

