# Supplementary material for: The Prevalence of Online Health Information Seeking Among Patients in Scotland: A Cross-Sectional Exploratory Study
Source: JMIR Res Protoc. 2015 Jul 15;4(3):e85. doi: 10.2196/resprot.4010 (PMC4526998; doi:10.2196/resprot.4010)
Supplement: Multimedia Appendix 1 [file resprot_v4i3e85_app1.pdf]

QUESTIONNAIRE ONLY TO BE COMPLETED IF YOU ARE 18 YEARS OLD OR MORE

- You are invited to take part in a survey to inform a student dissertation at the University of the Highlands and Islands in Scotland. This piece of research will enable a greater understanding of the impact that online health information has on frontline medical services. By completing this survey you will be aiding this understanding by giving information about your use of the internet for health information.

- No personal medical information is required. The nature of your illness is not something you will be asked to disclose here. The questionnaire is primarily concerned with whether you have consulted the internet prior to your visit, or if anyone has done so on your behalf. There will be a brief questionnaire which is designed to be completed as you wait for your appointment.

- The survey follows a strict confidentiality policy and if you choose to participate your responses will remain anonymous and shall not be personally disclosed to any other body. The survey is concerned with the general public as a whole and so individual responses will not be linked to participants in any way and shall be reported in aggregate form in any published research. The survey does NOT, at any point, ask for your name or date of birth. This survey adheres to the provision of the Data Protection Act 1998 and data which is gathered shall be stored securely for five years after which the data will be destroyed in a confidential manner.

- The questionnaire is entirely voluntary so please do not feel you have to take part. Completion of the survey indicates your consent and that you are over 18 years old. While your responses are appreciated we do not wish to cause any discomfort. If you do feel negatively affected by the questionnaire then please contact your doctor.

- If you wish to contact us regarding any aspect of the survey please do not hesitate to contact me via email or telephone, details of which are given below. Alternatively, you may contact my research supervisor, details of whom are given below.

Researcher:
Julia Moreland,
Department of Social Sciences,
UHI Moray College,
Moray Street,
Elgin, IV30 IJJ,
Tel. (0044)1343 576292

Research Supervisor:
Tara French,
Department of Social Sciences,
UHI Moray College,
Moray Street,
Elgin, IV30 IJJ,

Once completed please post the questionnaire into the box at reception and retain the information sheet (page 1).

1. Have you or someone acting on your behalf, previously used the internet to look up health information? (please circle)

Yes, myself Yes, someone on my behalf No Unknown

…..

If you answered "No" then please proceed to **1A** and answer any of the following questions which may apply to you. If you answered "Yes" then please proceed to **1B** and then continue with the rest of the questionnaire.

1A. If you do not use the Internet to get advice or information about health and health care, please indicate how concerned you are if at all, about these things. (Please circle indicating your level of concern)*

- **Your employer might find out what health sites you have gone to online.**

Concerned (very) *Concerned (somewhat)* *Concerned (not very)* *Not concerned* *Do not know/ Refused*

- **Getting health information from an unreliable source online.**

Concerned (very) *Concerned (somewhat)* *Concerned (not very)* *Not concerned* *Do not know/ Refused*

- **Other people might find out what health sites you have gone to online.**

Concerned (very) *Concerned (somewhat)* *Concerned (not very)* *Not concerned* *Do not know/ Refused*

- **A web site might sell or give away information about what you did online.**

Concerned (very) *Concerned (somewhat)* *Concerned (not very)* *Not concerned* *Do not know/ Refused*

…..

1B. Here are some things people say about going online to get advice or information about HEALTH and HEALTH CARE on the Internet. Thinking about the ways that you, personally,

might use the Internet to get information about health and health care, please select how important, if at all, each of these is to you? (Please circle indicating how important you feel these actions are)*

- **You can get health information online anonymously, without having to talk to anyone**.

Important (very) *Important (somewhat)* *Important (not very)* *Not important* *Do not know/ Refused*

- **You can get health information online any time, whenever it's convenient.**

Important (very) *Important (somewhat)* *Important (not very)* *Not important* *Do not know/ Refused*

- **You can get more health information online than from other sources.**

Important (very) *Important (somewhat)* *Important (not very)* *Not important* *Do not know/ Refused*

…..

2. How often do you use the Internet to look for advice or information about health or health care? (Please circle)*

Once a week *Once a month* *Every few months* *Less often* *Never* *Do not know/ Refused*

…..

3. Here are some things people sometimes do when getting advice or information about health or health care on the Internet. Some people have done these things, but other people have not. (Please circle all of the following you have done)*

a. Looked for information about a physical illness or condition that you or someone you know has.

b. Bought medicine or vitamins online.

c. Participated in an online support group for people who are concerned about the same health or medical issues.

d. Used email or gone to a web site to communicate with a health professional or their office.

e. Clicked on a health or medical web site's privacy policy to read about how the site uses personal information.

f. Described a medical condition or problem online in order to get advice from an online health professional.

g. Kept a health web site "bookmarked", or saved as a "favourite place", so you can go back to it regularly.

h. Looked to see what company or organisation is providing the advice or information that appears on a health website.

…..

4. Did you or someone acting on your behalf, search for health information recently with regard to your current appointment? (Please circle)

Yes, myself *Yes, someone on my behalf* *No* *Unknown*

……………………………………………………………………………………………………

5. If yes, did you or someone acting on your behalf use any of the following? (Please circle)

Health forum *Search engine* *NHS website* *Unknown*

Would you please provide the name of the websites if known?

____

……………………………………………………………………………………………………

6. Did the health information influence your decision to attend your appointment today? (Please circle)

Yes *No* *Unknown*

……………………………………………………………………………………………………

7. Would you have attended this medical centre today if you had not found this information? (Please circle)

Yes *No* *Unknown*

……………………………………………………………………………………………………

8. Have you previously found information on the internet which has helped you improve your health? (Please circle)

Yes, myself *Yes, someone on my behalf* *No* *Unknown*

……………………………………………………………………………………………………

9. Overall, how USEFUL was the health information you got online? (Please circle)*

Useful (very) *Useful (somewhat)* *Useful (not very)* *Not useful* *Do not know/ Refused*

……………………………………………………………………………………………………

10. Did you later talk to a health professional about the information you got online? (Please circle)*

Talked to a health professional *Did not talk to* *Do not know/Refused*

……………………………………………………………………………………………………

11. Did you learn anything NEW from the information you got online? (Please circle)*

Yes, learned something new *No, did not* *Do not know/Refused*

……………………………………………………………………………………………………

The following questions relate to characteristics of patients who use the internet:

12. What is your sex/gender? (Please circle) *Male* *Female*

……………………………………………………………………………………………………

13. What age group are you in?

18-25 *26-35* *36-45* *46-55*

56-65 *66-75* *76-85* *86+*

……

14. What is your ethnicity? (Please circle)

Asian *Black* *White* *Other*

……

15. What is your highest level of qualification? (Please circle)

No formal qualifications	*Standard Grade, O'Grades, O' levels, GCSE*	*A Levels, Highers, Advanced*	*Vocational Qualifications*		
		Degree	*Masters*	*Doctorate*	*Professional Qualification*
Other					

……

16. What is your current occupational status? (Please circle)

Full-time employment *Part-time employment* *Homemaker*

Self-employed *Full-time student* *Part-time student*

Unemployed *Retired* *Other*

*If other please state*____

……

17. Which of the following areas do you live in? (Please circle)

Rural *Village* *Town* *City*

……

18. Do you have internet access in your home? (Please circle)

Yes *No* *Unknown*

……

19. Is your internet connection broadband or dial up? (Please circle)

Broadband *Dial up* *Unknown*

……

20. Please circle which of the medical centres you are completing this questionnaire in:

GP Surgery *A&E Department* *Dentist* *Optician*

Many thanks for taking part in this survey. The information you have provided will help to increase what we know about internet use and how this affects our daily lives.

*Source - Fox, S. and Rainie, L. (2000) *The online health care revolution: How the Web helps Americans take better care of themselves* [online]. Washington: Pew Internet & American Life Project. Available from <http://www.pewinternet.org/Reports/2000/The-Online-Health-Care-Revolution.aspx> [8 December 2011]
